# Mutation of *neurotrophic tyrosine receptor kinase* can promote pan-cancer immunity and the efficacy of immunotherapy

**DOI:** 10.1186/s12943-024-01986-0

**Published:** 2024-04-25

**Authors:** Congren Wang, Yingying Li, Jinyuan Huang, Huimeng Yan, Bin Zhao

**Affiliations:** 1 Quanzhou First Hospital Affiliated to Fujian Medical University, Quanzhou, 362000 China; 2 Second Affiliated Hospital, Yuying Children’s Hospital, Wenzhou Medical University, Wenzhou, 325035 China

**Keywords:** Neurotrophic tyrosine receptor kinase, Immunotherapy, Cancer, Biomarker, Tumor microenvironment

## Abstract

The *Neurotrophic tyrosine receptor kinase (NTRK)* family plays important roles in tumor progression and is involved in tumor immunogenicity. Here, we conducted a comprehensive bioinformatic and clinical analysis to investigate the characteristics of *NTRK* mutations and their association with the outcomes in pan-cancer immunotherapy. In 3888 patients across 12 cancer types, patients with *NTRK*-mutant tumors showed more benefit from immunotherapy in terms of objective response rate (ORR; 41.7% vs. 27.5%; *P* < 0.001), progress-free survival (PFS; HR = 0.80; 95% CI, 0.68–0.96; *P* = 0.01), and overall survival (OS; HR = 0.71; 95% CI, 0.61–0.82; *P* < 0.001). We further constructed and validated a nomogram to estimate survival probabilities after the initiation of immunotherapy. Multi-omics analysis on intrinsic and extrinsic immune landscapes indicated that *NTRK* mutation was associated with enhanced tumor immunogenicity, enriched infiltration of immune cells, and improved immune responses. In summary, *NTRK* mutation may promote cancer immunity and indicate favorable outcomes in immunotherapy. Our results have implications for treatment decision-making and developing immunotherapy for personalized care.

**Supplementary Information:**

The online version contains supplementary material available at 10.1186/s12943-024-01986-0.

The application of immune checkpoint inhibitors (ICIs) targeting PD-1/PD-L1 and CTLA-4 has revolutionized cancer treatment in the past decade [1]. However, it is still difficult to determine which patients should be offered immunotherapy currently, and reliable biomarkers are needed [1, 2]. Mutations of *NTRK* genes are frequently detected in various tumors. They can trigger a number of signal pathways that regulate cell growth, proliferation, differentiation, apoptosis and survival [3], which may impact the tumor immunogenicity. Indeed, previous studies revealed that colorectal tumors harboring *NTRK* fusions defined a unique subtype with high microsatellite instability [4]. In lung cancer, *NTRK* alteration was positively associated high tumor mutation burden (TMB) [5]. We speculated the mutation of *NTRK* could enhance the immune responses and be a potential biomarker in immunotherapy. Therefore, here we conducted a comprehensive bioinformatic and clinical analysis to examine the characteristics of *NTRK* (*NTRK1, NTRK2*, and *NTRK3*) gene mutations and their association with the clinical outcomes of pan-cancer immunotherapy (Suppl. Methods).

Totally, 3888 patients from 14 datasets were included to examine the association between *NTRK* mutation and the efficacy of immunotherapy (Suppl. Table 1). The discovery cohort was an independent dataset enrolled 1610 patients with 10 cancer types, including lung cancer (*n* = 344), melanoma (*n* = 314), bladder urothelial cancer (*n* = 211), renal cancer (*n* = 143), head and neck cancer (*n* = 129), esophagogastric cancer (*n* = 118), glioma (*n* = 116), colorectal cancer (*n* = 109), cancer of unknown primary (*n* = 85), breast cancer (*n* = 41). Compared with *NTRK* non-mutation, patients with *NTRK*-mutated tumors achieved favorable OS (HR = 0.63; 95% CI, 0.50–0.80; *P* < 0.001; Fig. 1A). 2278 patients with 7 tumor types from 13 datasets were pooled into the validation cohort. These patients were diagnosed as lung cancer (*n* = 902), renal cancer (*n* = 760), melanoma (*n* = 575), bladder urothelial cancer (*n* = 27), head and neck cancer (*n* = 12), sarcoma (*n* = 1), and anal cancer (*n* = 1). *NTRK* mutation was also associated with longer OS (HR = 0.80; 95% CI, 0.65–0.97; *P* = 0.03; Fig. 1B). Overall, in 3888 patients with 12 cancer types who were treated with ICIs, *NTRK* mutation (*n* = 465) decreased the risk of death by 29% (HR = 0.71; 95% CI, 0.61–0.82; *P* < 0.001; Fig. 1C). Additionally, patients with *NTRK* mutation showed better ORR (41.7% vs. 27.5%; *P* < 0.001; Fig. 1D) and PFS (HR = 0.80; 95% CI, 0.68–0.96; *P* = 0.01; Fig. 1E). Specifically, *NTRK3* mutations were discovered in 229 patients and associated with robust anti-cancer activities in terms of ORR (45.4% vs. 28.3%; *P* < 0.001), PFS (HR = 0.72; 95% CI, 0.58–0.89; *P* = 0.01), and OS (HR = 0.60; 95% CI, 0.50–0.73; *P* < 0.001) (Suppl Fig. 1). *NTRK2* mutation (*n* = 105) predicted similar outcomes but to a lesser extent in ORR (40.7% vs. 29.1%; *P* = 0.05), PFS (HR = 0.70; 95% CI, 0.51–0.95; *P* = 0.05), and OS (HR = 0.58; 95% CI, 0.45–0.76; *P* = 0.001). Of note, the predive performances of *NTRK1* mutation (*n* = 188) were only marginal in ORR (38.9% vs. 28.9%; *P* = 0.02), PFS (HR = 0.87; 95% CI, 0.69–1.09; *P* = 0.25), and OS (HR = 0.80; 95% CI, 0.63–1.02; *P* = 0.09).

Both univariate (Fig. 1F) and multivariate (Fig. 1G) Cox analysis confirmed that *NTRK* mutation was an independent biomarker for OS (HR = 0.83; 95% CI, 0.69–0.99; *P* = 0.04) and PFS (HR = 0.77; 95% CI, 0.65–0.92; *P* = 0.004) (Suppl Fig. 2). Hence, we developed a nomogram to estimate 12-month and 24-month OS after the initiation of immunotherapy based on the discovery cohort (Fig. 1H). Further analysis on the calibrations of these predictions suggested this cure-model-based nomogram was good (Suppl Fig. 3). The optimal cutoff value (total points = 130) determined by X-tile software was introduced and categorized patients into high-score and low-score subgroups. Low-score was associated with favorable OS in both discovery cohort (HR = 0.48; 95% CI, 0.41–0.55; *P* < 0.001; Fig. 1I) and validation cohort (HR = 0.76; 95% CI, 0.64–0.90; *P* = 0.001; Fig. 1J).

**Fig. 1:**
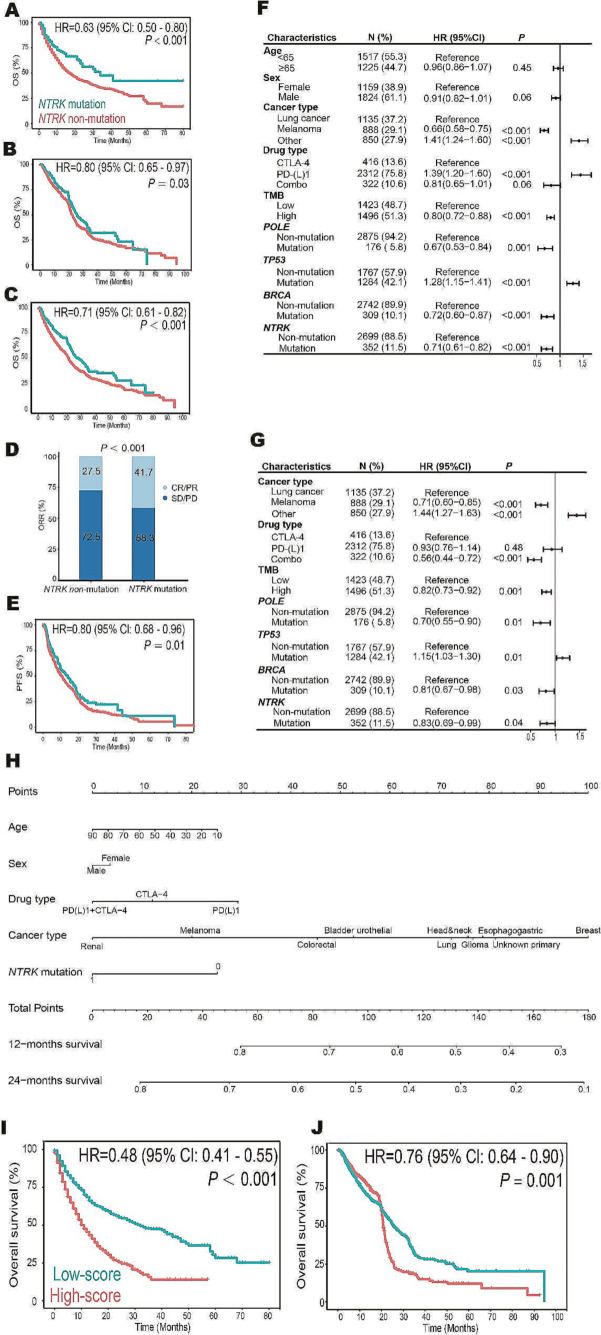
The mutation of *NTRK* gene family as an independent predictive biomarker in pan-cancer immunotherapy. (**A**) Kaplan–Meier survival analysis stratified by *NTRK* mutation status in 1610 cancer patients with 10 types of tumors treated with ICIs in the discovery cohort. (**B**) Association between *NTRK* mutation and OS in 2278 patients with 7 types of tumors treated with ICIs in the validation cohort. (**C**-**E**) Comparison of OS (**C**), ORR (**D**), and PFS (**E**) between patients with *NTRK* mutation and patients with *NTRK* non-mutation in 3888 patients with 12 tumor types treated with ICIs. (**F**-**G**) Univariate (**F**) and multivariate (**H**) Cox analysis of the association between *NTRK* mutation and OS in 3888 patients with 12 tumor types treated with ICIs. (**H**) Nomogram to predict the 12- and 24-month survival. It can calculate overall survival from the date of immunotherapy start. To use, locate ‘age’ axis and draw a line up to the ‘point’ axis to get a score associated with age, repeat for the other features to get their scores. Sum all scores and locate it on the ‘total point’ axis, draw a line to ’12-month survival’ axis to get the 12-month OS probability. (**I**-**J**) Based on the optimal cutoff value derived from nomogram, low-score was associated with favorable OS in both discovery cohort (**I**) and validation cohort (**J**). CI, confidence interval; CR, complete response; HR, hazard ratio; ICI, immune checkpoint inhibitor; ORR, objective response rate; OS, overall survival; PD, progressive disease; PFS, progression-free survival; PR, partial response; SD, stable disease; TMB, tumor mutation burden

To explore the underlying mechanisms between *NTRK* mutation and cancer immunotherapy, multi-omics information extracted from the cancer genome atlas (TCGA) cohort were investigated to reveal the tumor immune microenvironment. We first explored the somatic mutant frequencies of three *NTRK* genes in TCGA pan-cancer cohort. 568 of all 10,953 enrolled patients (5.19%) harbored *NTRK* mutations. They were found in a small subset of most types of tumors (Suppl. Figure 4), and the mutant frequencies differed significantly among various tumors (*P* < 0.001). Specifically, *NTRK3* mutations were observed in 292 patients (2.67%), *NTRK1* in 187 patients (1.71%) and *NTRK2* in 170 patients (1.55%). Totally, 733 *NTRK* mutations were identified (Suppl. Table 1), 606 (82.7%) were missense mutations, 49 (6.7%) were truncating mutations, 38 (5.2%) were spice mutations, 38 (5.2%) were fusion mutations, and 2 (0.3%) were inframe mutations. Moreover, the prognosis for cancer patients were independent of *NTRK* mutations in terms of OS (HR = 1.09; 95% CI, 0.94–1.27; *P* = 0.23) and PFS (HR = 1.06; 95% CI, 0.92–1.22; *P* = 0.45) (Suppl. Figure 5).

The major intrinsic immune response included high tumor immunogenicity, activation of the antigen-processing machinery, and the over-expression of costimulatory molecules [6]. As shown in Fig. 2A, *NTRK* mutation was associated with higher TMB, non-silent mutation rate, and silent mutation rate. Next, we examined if there were any specific mutation patterns which were associated with the efficacy of immunotherapy. The frequencies of all known COSMIC reference signatures in *NTRK*-mutant and *NTRK*-non-mutant tumors were compared. As shown in Suppl Fig. 6A, the frequencies of SBS7a (known etiology, ultraviolet light exposure), SBS10b (*POLE* mutation), SBS30 (defective DNA base excision repair), and SBS86 (unknown chemotherapy treatment) changed significantly in *NTRK*-mutant tumors. Further analysis revealed these four signatures were predictive biomarkers for OS in patients treated with ICIs (Suppl Fig. 6B). Additionally, the mRNA expression levels of three major immune checkpoints (*PD-1*, *PD-L1*, and *CTLA-4*) were significantly elevated in *NTRK*-mutant tumors (Fig. 2B). We also observed most of 16 major histocompatibility complex (MHC) and 25 costimulatory molecules were increased in *NTRK*-mutant tumors (Fig. 2G).

**Fig. 2:**
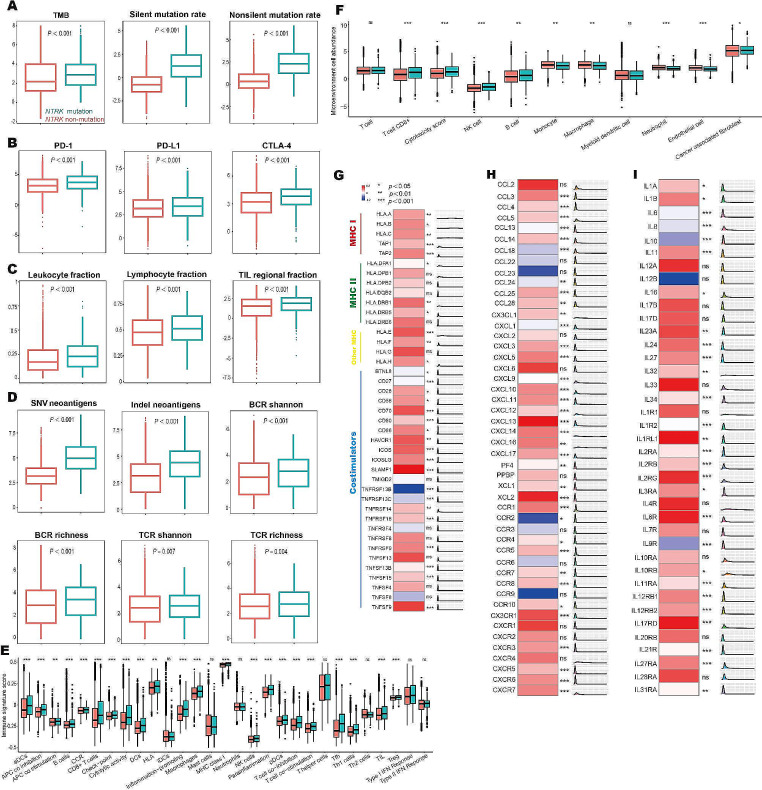
The characteristics of tumor immune microenvironment in patients with *NTRK*-mutant and *NTRK* -non-mutant cancer. (A) Comparison of TMB, non-silent mutation rate, and silent mutation rate between *NTRK-*mutant and *NTRK*-non-mutant tumors. (B) Expression of three major immune checkpoints in patients with *NTRK-*mutant and *NTRK-*non-mutant tumors. (C) The immune cell infiltration revealed by leukocyte fractions, lymphocytes fraction and tumor-infiltrating lymphocyte fraction in *NTRK-*mutant and *NTRK*-non-mutant tumors. (D) The abundances of SNV /Indel neoantigens and the diversity of TCR/BCR in *NTRK-*mutant and *NTRK*-non-mutant tumors. (E) Differences of 29 immune signatures estimated by ssGSEA between *NTRK-*mutant and *NTRK-*non-mutant tumors. (F) Comparison of 9 immune and 2 stromal cell populations between *NTRK-*mutant and *NTRK-*non-mutant tumors. (G) Expression differences of 16 MHC-related antigen-presenting molecules and 25 co-stimulators between *NTRK-*mutant and *NTRK-*non-mutant tumors. (H) Comparison of 48 chemokines and their receptors between *NTRK-*mutant and *NTRK-*non-mutant tumors. (I) Expression differences of 39 immune-stimulators between *NTRK-*mutant and *NTRK-*non-mutant tumors. BCR, B cell receptor; CTLA-4, cytotoxic T-lymphocyte-associated antigen 4; MHC, major histocompatibility complex; PD-1, programmed cell death protein 1; PD-L1, programmed cell death ligand 1; SNV, single nucleotide variants; TCR, T cell receptor; TIL, tumor-infiltrating lymphocyte; TMB, tumor mutation burden

The key extrinsic immune characteristics included the infiltration of immune cells into the tumor microenvironment, high diversity of B cell receptors (BCRs) and T cell receptors (TCRs), activated immunogenicity of cancer cells contribute to the immune response, and high expression level of immune-stimulators and chemokines [7]. Compared with *NTRK*-non-mutant tumors (Fig. 2C), *NTRK*-mutant tumors exhibited higher levels of immune cell infiltration according to (1) leukocyte fractions measured by DNA methylation arrays; (2) lymphocytes fraction estimated from CIBERSORT algorithm; and (3) genomic evaluation of the tumor-infiltrating lymphocyte (TIL) fraction. The abundances of SNV/Indel neoantigens and the diversity of TCR/BCR were significantly upregulated in *NTRK*-mutant tumors (Fig. 2D). ssGSEA could quantify 29 common immune signatures including key immune pathways, cells, and functions in tumor microenvironment (Fig. 2E) [8]. The MCP-counter method calculated the abundance of 9 immune and 2 stromal cell populations (Fig. 2F) [9]. The immune signatures and cell populations were clearly enriched in *NTRK*-mutant tumors. Additionally, *NTRK*-mutant tumors were associated with increased expression of 48 known chemokines and their receptors (Fig. 2H) and 39 immune-stimulators (Fig. 2I).

These results derived from intrinsic and extrinsic immune landscapes indicated that *NTRK* mutation was associated with enhanced tumor immunogenicity, enriched infiltration of immune cells, and improved immune responses, which might explain that patients with *NTRK* mutant tumors showed favorable outcomes when treated with ICIs.

In summary, *NTRK-*mutant tumors might be regarded as immunologically “hot” tumors as they could promote both intrinsic and extrinsic tumor immunogenicity. Moreover, *NTRK* mutation was an independent biomarker for favorable outcomes in cancer immunotherapy. These results have implications for treatment decision-making and developing immunotherapy for personalized care.

### Electronic supplementary material

Below is the link to the electronic supplementary material.

Supplementary Material 1

Supplementary Material 2

Supplementary Material 3

Supplementary Material 4

Supplementary Material 5

Supplementary Material 6

Supplementary Material 7

Supplementary Material 8

Supplementary Material 9

## Data Availability

The datasets generated during and/or analyzed during the current study are available from the corresponding author upon reasonable request.

## Abbreviations

BCR: B cell receptor
CI: confidence interval
CR: complete response
CTLA-4: cytotoxic T-lymphocyte-associated antigen 4
HR: hazard ratio
ICI: immune checkpoint inhibitor
MHC: major histocompatibility complex
NTRK: neurotrophic tyrosine receptor kinase
ORR: objective response rate
OS: overall survival
PD: progressive disease
PD-1: programmed cell death protein 1
PD-L1: programmed cell death ligand 1
PFS: progression-free survival
PR: partial response
SD: stable disease
SNV: single nucleotide variants
TCR: T cell receptor
TIL: tumor-infiltrating lymphocyte
TMB: tumor mutation burden
