# Supplementary figures and images for: Mutation of neurotrophic tyrosine receptor kinase can promote pan-cancer immunity and the efficacy of immunotherapy
Source: Mol Cancer. 2024 Apr 25;23:81. doi: 10.1186/s12943-024-01986-0 (PMC11044367; doi:10.1186/s12943-024-01986-0)

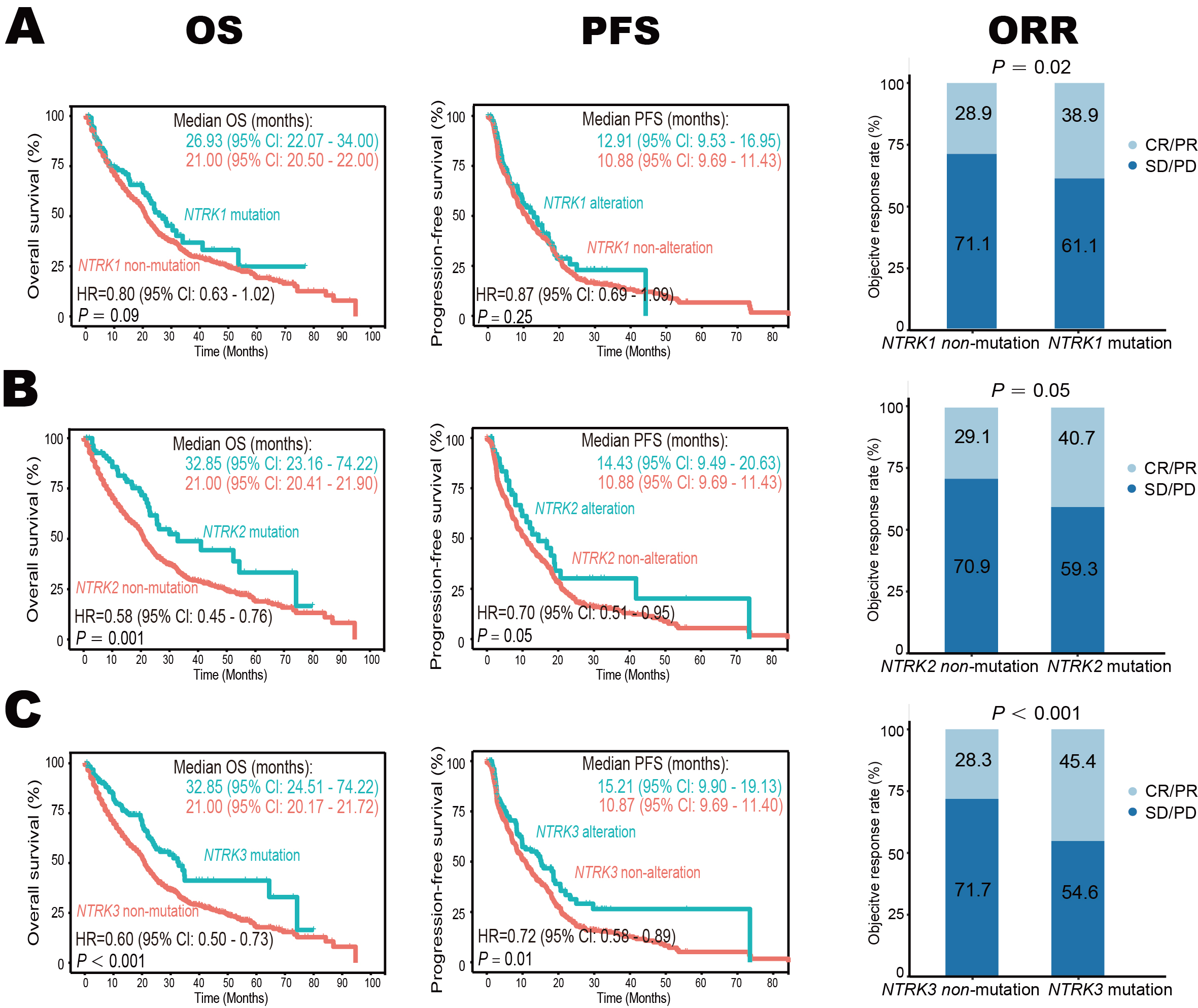

Supplement: Supplementary file 1 — Supplementary Material 1 [file 12943_2024_1986_MOESM1_ESM.jpg]

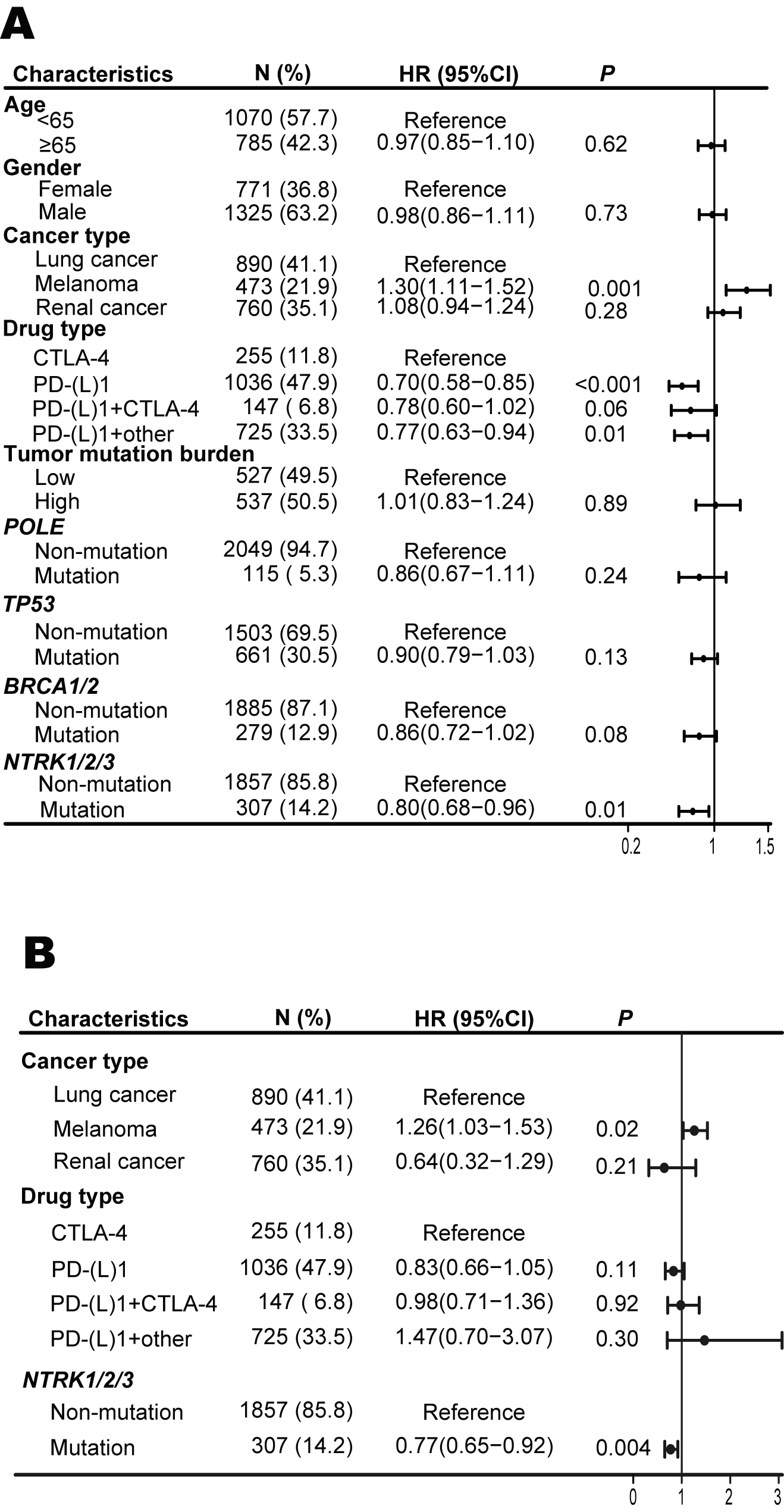

Supplement: Supplementary file 2 — Supplementary Material 2 [file 12943_2024_1986_MOESM2_ESM.jpg]

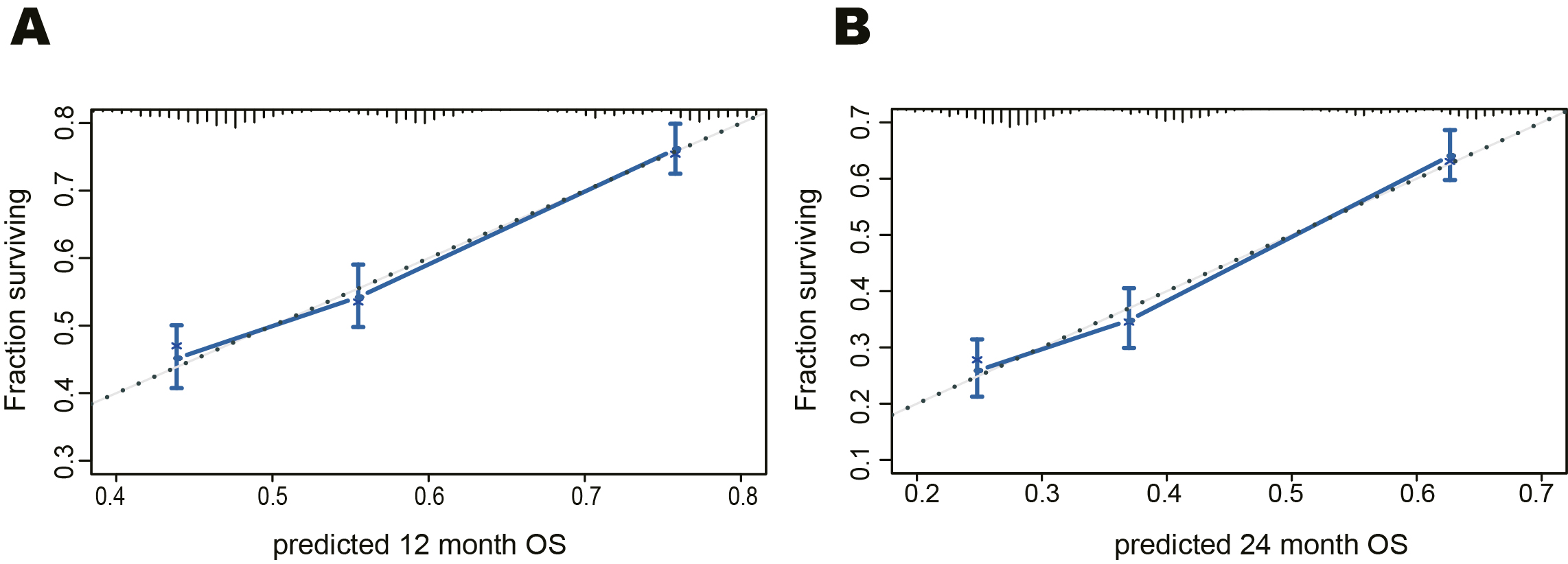

Supplement: Supplementary file 3 — Supplementary Material 3 [file 12943_2024_1986_MOESM3_ESM.jpg]

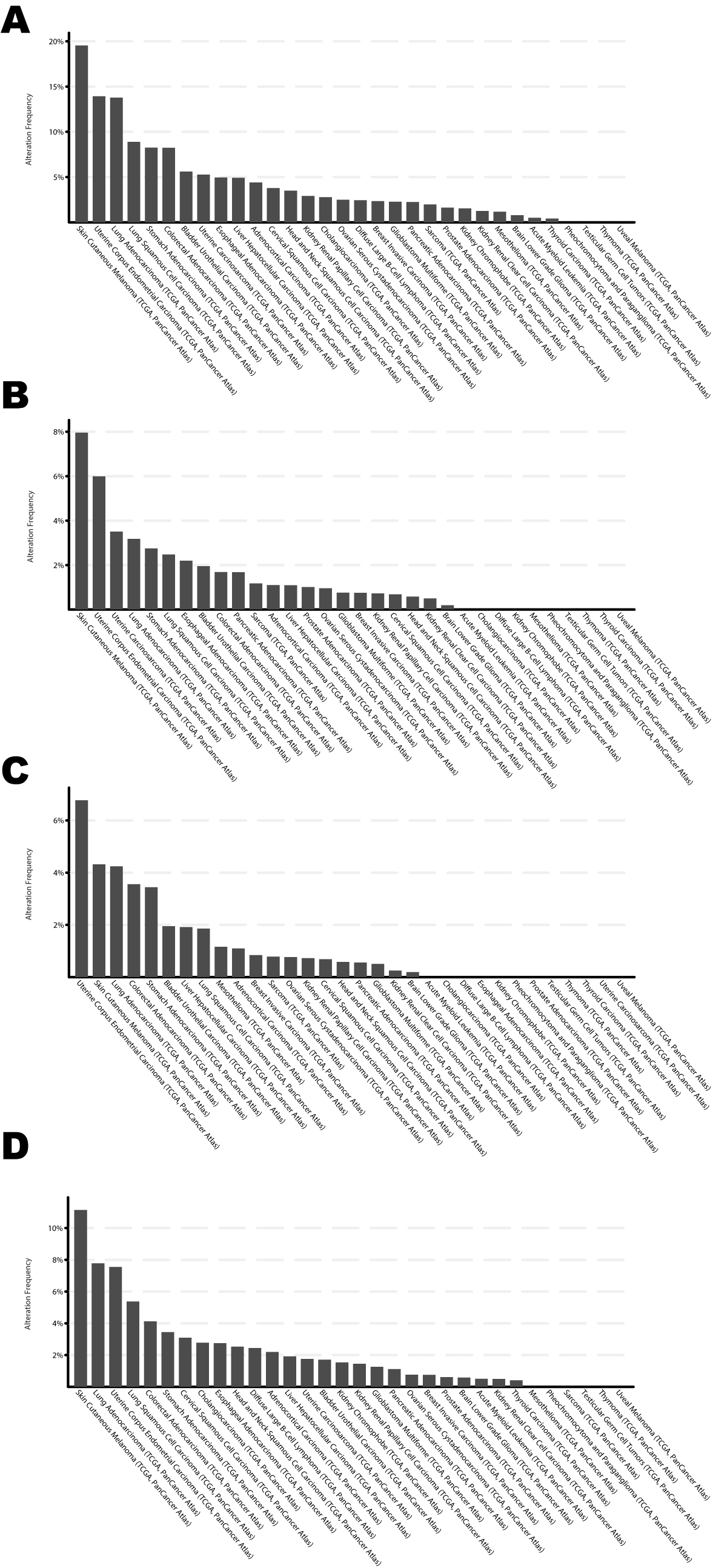

Supplement: Supplementary file 4 — Supplementary Material 4 [file 12943_2024_1986_MOESM4_ESM.jpg]

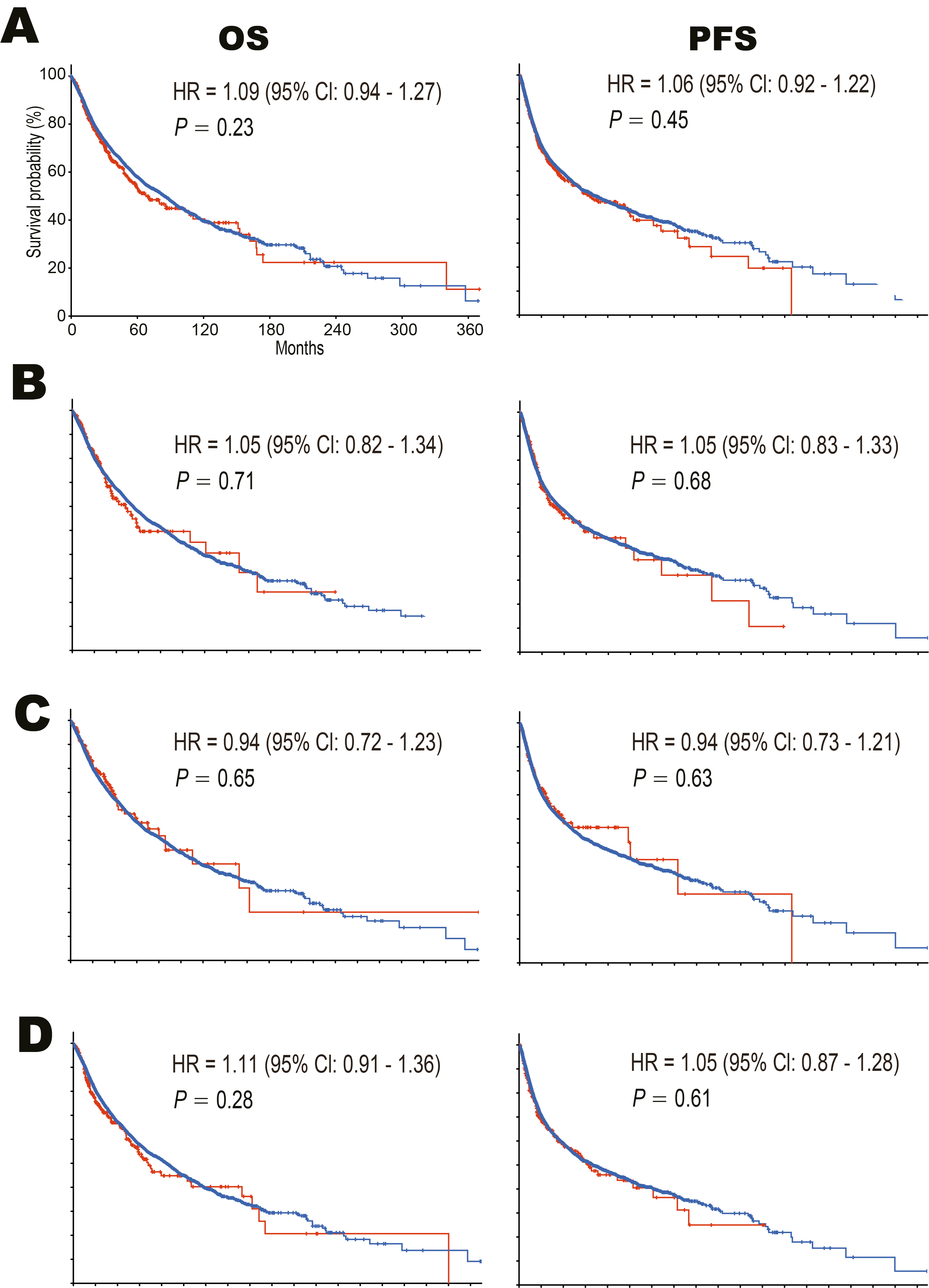

Supplement: Supplementary file 5 — Supplementary Material 5 [file 12943_2024_1986_MOESM5_ESM.jpg]

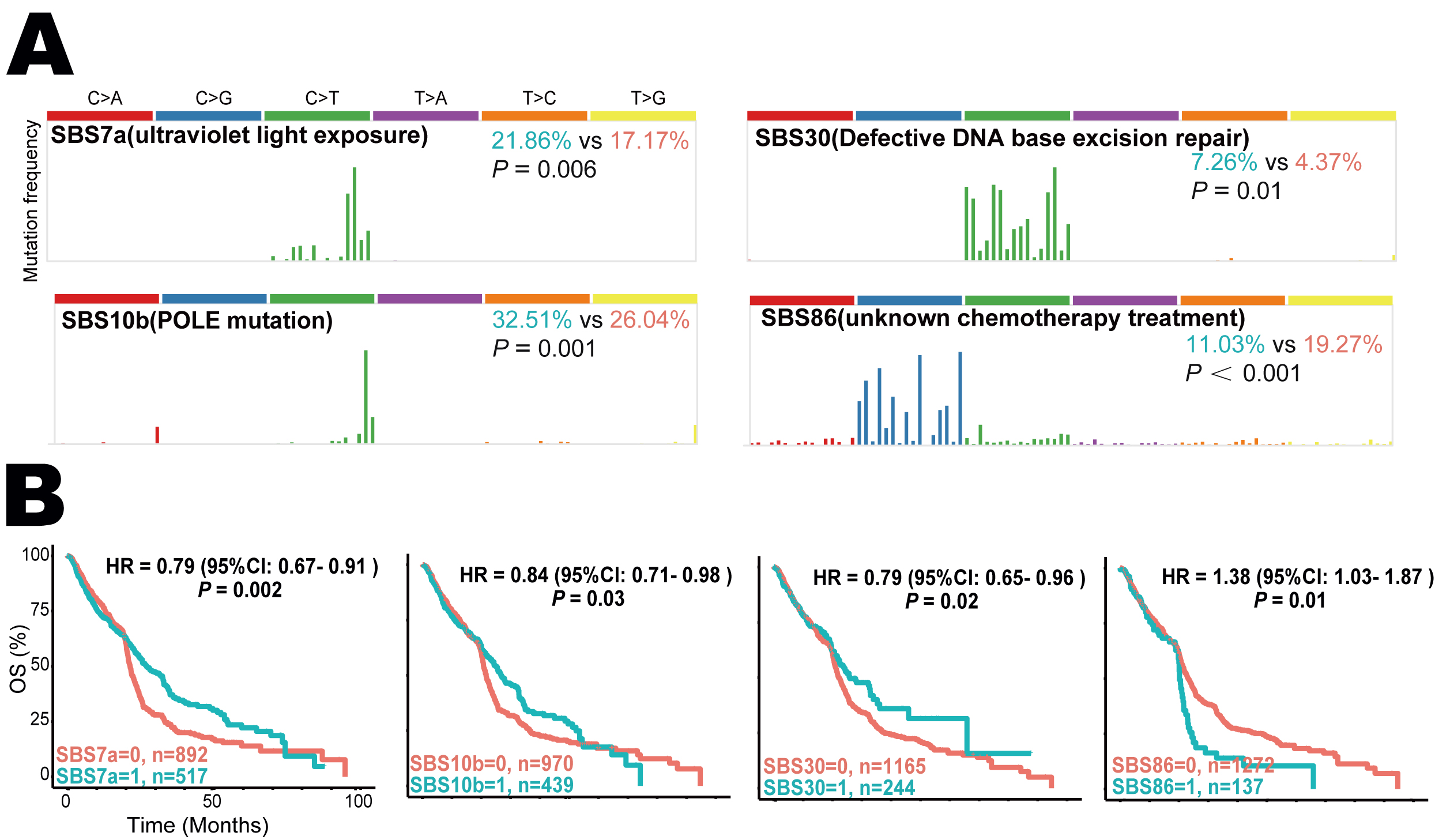

Supplement: Supplementary file 6 — Supplementary Material 6 [file 12943_2024_1986_MOESM6_ESM.jpg]
