# Supplementary material for: Mutation of neurotrophic tyrosine receptor kinase can promote pan-cancer immunity and the efficacy of immunotherapy
Source: Mol Cancer. 2024 Apr 25;23:81. doi: 10.1186/s12943-024-01986-0 (PMC11044367; doi:10.1186/s12943-024-01986-0)
Supplement: Supplementary file 7 — Supplementary Material 7 [file 12943_2024_1986_MOESM7_ESM.docx]

**Suppl. Table 1.** Baseline characteristics of the eligible studies for cancer immunotherapy analysis.

| **Author, year** | **Treatment agents** | **Cancer type** | ***NTRK* mutation** | **No. of patients** | **No. of responses** |
| --- | --- | --- | --- | --- | --- |
| **Discovery cohort** | | | | | |
| Samstein, 2019 [1] | Inhibitors targeting CTLA-4, PD-1, and PD-L1 | Multiple tumors | Positive | 143 | 7 |
|  |  |  | Negative | 1467 | 45 |
| **Validation cohort** | | | | | |
| Hugo, 2016 [2] | Pembrolizumab/Nivolumab | Melanoma | Positive | 5 | 1 |
|  |  |  | Negative | 33 | 20 |
| Liu, 2019 [3] | Pembrolizumab/Nivolumab | Melanoma | Positive | 31 | 15 |
|  |  |  | Negative | 113 | 40 |
| Miao, 2018 [4] | Inhibitors targeting CTLA-4, PD-1, and PD-L1 | Multiple tumors | Positive | 46 | 19 |
|  |  |  | Negative | 203 | 51 |
| Riaz, 2017 [5] | Nivolumab | Melanoma | Positive | 5 | 3 |
|  |  |  | Negative | 63 | 12 |
| Van Allen, 2015 [6] | Ipilimumab | Melanoma | Positive | 16 | 6 |
|  |  |  | Negative | 94 | 11 |
| Gandara, 2018 [7] | Atezolizumab | Lung cancer | Positive | 58 | 15 |
|  |  |  | Negative | 369 | 47 |
| Ravi, 2023 [8] | Inhibitors targeting CTLA-4 and PD-1 or in combination with chemotherapy | Lung cancer | Positive | 40 | 20 |
|  |  |  | Negative | 269 | 101 |
| Snyder, 2014 [9] | Ipilimumab/Tremelimumab | Melanoma | Positive | 8 | NA |
|  |  |  | Negative | 56 | NA |
| Hellmann, 2018 [10] | Inhibitors targeting CTLA-4 and PD-1 | Lung cancer | Positive | 20 | 7 |
|  |  |  | Negative | 55 | 17 |
| Miao, 2018 [11] | Nivolumab | Renal cancer | Positive | 1 | 1 |
|  |  |  | Negative | 34 | 6 |
| Motzer, 2020 [12] | Atezolizumab + bevacizumab | Renal cancer | Positive | 42 | 21 |
|  |  |  | Negative | 317 | 118 |
| Motzer, 2020 [13] | Avelumab + axitinib | Renal cancer | Positive | 44 | NA |
|  |  |  | Negative | 322 | NA |
| Rizvi, 2015 [14] | Pembrolizumab | Lung cancer | Positive | 6 | 3 |
|  |  |  | Negative | 28 | 9 |

NA, not available

**Suppl Table 2.** Different subtypes of *NTRK* mutations in pan-cancer TCGA cohort.

| **Gene** | **No. of total mutations (%)** | **No. of missense mutations (%)** | **No. of truncating mutations (%)** | **No. of spice mutations (%)** | **No. of fusion mutations (%)** | **No. of Inframe mutations (%)** |
| --- | --- | --- | --- | --- | --- | --- |
| *NTRK* | 733 (100%) | 606 (82.7%) | 49 (6.7%) | 38 (5.2%) | 38 (5.2%) | 2 (0.3%) |
| *NTRK1* | 211 (100%) | 174 (82.5%) | 8 (3.8%) | 14 (6.6%) | 14 (6.6%) | 1 (0.5%) |
| *NTRK2* | 183 (100%) | 152 (83.1%) | 16 (8.7%) | 8 (4.4%) | 6 (3.3%) | 1 (0.5%) |
| *NTRK3* | 339 (100%) | 280 (82.6%) | 25 (7.4%) | 16 (4.7%) | 18 (5.3%) | 0 (0.0%) |

**References:**

1. Samstein, R.M., et al., *Tumor mutational load predicts survival after immunotherapy across multiple cancer types.* Nat Genet, 2019. **51**(2): p. 202-206.

2. Hugo, W., et al., *Genomic and Transcriptomic Features of Response to Anti-PD-1 Therapy in Metastatic Melanoma.* Cell, 2016. **165**(1): p. 35-44.

3. Liu, D., et al., *Integrative molecular and clinical modeling of clinical outcomes to PD1 blockade in patients with metastatic melanoma.* Nat Med, 2019. **25**(12): p. 1916-1927.

4. Miao, D., et al., *Genomic correlates of response to immune checkpoint blockade in microsatellite-stable solid tumors.* Nat Genet, 2018. **50**(9): p. 1271-1281.

5. Riaz, N., et al., *Tumor and Microenvironment Evolution during Immunotherapy with Nivolumab.* Cell, 2017. **171**(4): p. 934-949 e16.

6. Van Allen, E.M., et al., *Genomic correlates of response to CTLA-4 blockade in metastatic melanoma.* Science, 2015. **350**(6257): p. 207-211.

7. Gandara, D.R., et al., *Blood-based tumor mutational burden as a predictor of clinical benefit in non-small-cell lung cancer patients treated with atezolizumab.* Nat Med, 2018. **24**(9): p. 1441-1448.

8. Ravi, A., et al., *Genomic and transcriptomic analysis of checkpoint blockade response in advanced non-small cell lung cancer.* Nat Genet, 2023. **55**(5): p. 807-819.

9. Snyder, A., et al., *Genetic basis for clinical response to CTLA-4 blockade in melanoma.* N Engl J Med, 2014. **371**(23): p. 2189-2199.

10. Hellmann, M.D., et al., *Genomic Features of Response to Combination Immunotherapy in Patients with Advanced Non-Small-Cell Lung Cancer.* Cancer Cell, 2018. **33**(5): p. 843-852.e4.

11. Miao, D., et al., *Genomic correlates of response to immune checkpoint therapies in clear cell renal cell carcinoma.* Science, 2018. **359**(6377): p. 801-806.

12. Motzer, R.J., et al., *Molecular Subsets in Renal Cancer Determine Outcome to Checkpoint and Angiogenesis Blockade.* Cancer Cell, 2020. **38**(6): p. 803-817.e4.

13. Motzer, R.J., et al., *Avelumab plus axitinib versus sunitinib in advanced renal cell carcinoma: biomarker analysis of the phase 3 JAVELIN Renal 101 trial.* Nat Med, 2020. **26**(11): p. 1733-1741.

14. Rizvi, N.A., et al., *Cancer immunology. Mutational landscape determines sensitivity to PD-1 blockade in non-small cell lung cancer.* Science, 2015. **348**(6230): p. 124-8.
