# Supplementary material for: Mutation of neurotrophic tyrosine receptor kinase can promote pan-cancer immunity and the efficacy of immunotherapy
Source: Mol Cancer. 2024 Apr 25;23:81. doi: 10.1186/s12943-024-01986-0 (PMC11044367; doi:10.1186/s12943-024-01986-0)
Supplement: Supplementary file 8 — Supplementary Material 8 [file 12943_2024_1986_MOESM8_ESM.docx]

**Suppl Methods**

**Data collection**

Patients with *NTRK-*mutant tumor who were treated with ICIs were extracted from 14 studies ^1-14^ (Suppl. Table 1). Among them, melanoma was investigated in 5 studies, lung cancer in 4 studies, renal cancer in 3 studies, and multiple tumors in 2 studies. Whole-exome sequencing (WES) was applied for sequencing in 12 cohorts ^1-10,12,13^. MSK-IMPACT panel was employed in one study with multiple tumors ^14^, and Foundation One assay was used in one study with renal cancer^11^. Agents targeting CTLA-4 were applied in 6 studies, inhibitors targeting PD-1/PD-L1 were administrated in 12 studies. Objective response rate (ORR), Progression-free survival (PFS) and overall survival (OS) were collected directly from the original reports. Patients who showed complete response (CR) or partial response (PR) were categorized as responders; patients who experienced stable disease (SD) or progressive disease (PD) were classified as non-responders.

Information regarding DNA methylation and sequencing, RNA expression, and clinicopathological characteristics of the cancer genome atlas (TCGA) cohort, were downloaded from https://gdc.cancer.gov/about-data/publications/pancanatlas. Key features used in this study (including silent mutation rate, non-silent mutation rate, SNV neoantigen, indel neoantigen, lymphocyte fraction, leukocyte fraction, CD8 T cell abundance, TIL regional fraction, TCR richness, and TCR Shannon index) were calculated as previously described ^15^.

**Statistics**

Survival curves were generated by Kaplan-Meier method and the log-rank test was used to evaluate the significance of differences. Hazard ratio (HR) and its 95% confidence interval (CI) was calculated by Cox proportional hazards model. Kruskal-Wallis, Wilcoxon test, and Chi-square test was used to analyze the associations among various categorical variables depend on the context. ssGSEA^16^ and MCP-counter analysis^17^ were conducted to examine tumor immune micro-environment. The ‘rms’ R package was used to generate both nomogram and calibration curves. The ‘deconstructSigs’ R package was applied to perform non-negative matrix factorization analysis of mutations and patterns of carcinoma evolution^18^. All data processing and analysis were carried out by R software (version 4.2.1) and MedCalc (version18.2.1). Two-sided *P* <0.05 were considered statistically significant.

**References:**

1. Hugo W, Zaretsky JM, Sun L, et al. Genomic and Transcriptomic Features of Response to Anti-PD-1 Therapy in Metastatic Melanoma. *Cell* 2016; **165**(1): 35-44.

2. Liu D, Schilling B, Liu D, et al. Integrative molecular and clinical modeling of clinical outcomes to PD1 blockade in patients with metastatic melanoma. *Nature medicine* 2019; **25**(12): 1916-27.

3. Miao D, Margolis CA, Vokes NI, et al. Genomic correlates of response to immune checkpoint blockade in microsatellite-stable solid tumors. *Nature genetics* 2018; **50**(9): 1271-81.

4. Riaz N, Havel JJ, Makarov V, et al. Tumor and Microenvironment Evolution during Immunotherapy with Nivolumab. *Cell* 2017; **171**(4): 934-49.e16.

5. Van Allen EM, Miao D, Schilling B, et al. Genomic correlates of response to CTLA-4 blockade in metastatic melanoma. *Science (New York, NY)* 2015; **350**(6257): 207-11.

6. Gandara DR, Paul SM, Kowanetz M, et al. Blood-based tumor mutational burden as a predictor of clinical benefit in non-small-cell lung cancer patients treated with atezolizumab. *Nature medicine* 2018; **24**(9): 1441-8.

7. Ravi A, Hellmann MD, Arniella MB, et al. Genomic and transcriptomic analysis of checkpoint blockade response in advanced non-small cell lung cancer. *Nat Genet* 2023; **55**(5): 807-19.

8. Snyder A, Makarov V, Merghoub T, et al. Genetic basis for clinical response to CTLA-4 blockade in melanoma. *N Engl J Med* 2014; **371**(23): 2189-99.

9. Hellmann MD, Nathanson T, Rizvi H, et al. Genomic Features of Response to Combination Immunotherapy in Patients with Advanced Non-Small-Cell Lung Cancer. *Cancer cell* 2018; **33**(5): 843-52.e4.

10. Miao D, Margolis CA. Genomic correlates of response to immune checkpoint therapies in clear cell renal cell carcinoma. *Science (New York, NY)* 2018; **359**(6377): 801-6.

11. Motzer RJ, Banchereau R, Hamidi H, et al. Molecular Subsets in Renal Cancer Determine Outcome to Checkpoint and Angiogenesis Blockade. *Cancer cell* 2020; **38**(6): 803-17.e4.

12. Motzer RJ, Robbins PB, Powles T, et al. Avelumab plus axitinib versus sunitinib in advanced renal cell carcinoma: biomarker analysis of the phase 3 JAVELIN Renal 101 trial. *Nature medicine* 2020; **26**(11): 1733-41.

13. Rizvi NA, Hellmann MD, Snyder A, et al. Cancer immunology. Mutational landscape determines sensitivity to PD-1 blockade in non-small cell lung cancer. *Science (New York, NY)* 2015; **348**(6230): 124-8.

14. Samstein RM, Lee C-H, Shoushtari AN, et al. Tumor mutational load predicts survival after immunotherapy across multiple cancer types. *Nature genetics* 2019; **51**(2): 202-6.

15. Thorsson V, Gibbs DL, Brown SD, et al. The Immune Landscape of Cancer. *Immunity* 2018; **48**(4): 812-30.e14.

16. Hänzelmann S, Castelo R, Guinney J. GSVA: gene set variation analysis for microarray and RNA-seq data. *BMC bioinformatics* 2013; **14**: 7.

17. Becht E, Giraldo NA, Lacroix L, et al. Estimating the population abundance of tissue-infiltrating immune and stromal cell populations using gene expression. *Genome biology* 2016; **17**(1): 218.

18. Rosenthal R, McGranahan N, Herrero J, Taylor BS, Swanton C. DeconstructSigs: delineating mutational processes in single tumors distinguishes DNA repair deficiencies and patterns of carcinoma evolution. *Genome biology* 2016; **17**: 31.
