# Supplementary material for: Mutation of neurotrophic tyrosine receptor kinase can promote pan-cancer immunity and the efficacy of immunotherapy
Source: Mol Cancer. 2024 Apr 25;23:81. doi: 10.1186/s12943-024-01986-0 (PMC11044367; doi:10.1186/s12943-024-01986-0)
Supplement: Supplementary file 9 — Supplementary Material 9 [file 12943_2024_1986_MOESM9_ESM.docx]

**Suppl. Figure 1.**

The association between *NTRK* mutation and the efficacies of cancer immunotherapy in term of OS (left panel), PFS (middle panel), and ORR (right panel) in 3888 patients with 12 types of tumors.

1. *NTRK1*; (B) *NTRK2*; and (C) *NTRK3*

**Suppl. Figure 2.**

Univariate (A) and multivariate Cox analysis (B) to evaluate the performance of various features as predictors for PFS in 3888 cancer patients treated with ICIs.

**Suppl Figure 3.**

Calibration plots for validation of the 12- month (A) and 24-month (B) survival from the nomogram in the discovery cohort with 1610 cancer patients. The average predicted probability (X axis) was plotted against the observed Kaplan-Meier estimate in the subgroup (Y axis, 95% CIs of the estimates are presented as vertical lines). Continuous line is the reference line, indicating what an optimal nomogram would be.

**Suppl. Figure 4.**

The mutation frequencies of *NTRK* gene family (A), *NTRK1* (B), *NTRK2* (C), and *NTRK3* (D) in 33 types of tumors based on TCGA pan-cancer cohort.

**Suppl. Figure 5.**

The association between *NTRK* mutation status and OS (left panel) or PFS (right panel) revealed by K-M survival curves in TCGA pan-cancer cohort.

1. *NTRK* gene family; (B) *NTRK1*; (C) *NTRK2*; and (D) *NTRK3*

**Suppl Figure 6.**

COSMIC reference signatures associated with *NTRK* mutation

1. The illustrations of four identified SBS signatures related with *NTRK* mutation and their frequencies in *NTRK*-mutant and *NTRK*-non-mutant tumors.

Bold black, SBS signature and its known etiologies. Green, frequency in *NTRK*-mutant cancer. Orange, frequency in *NTRK*-non-mutant cancer.

1. The associations between four identified mutation signatures with OS in cancer immunotherapy.

HR, hazard ratio, OS, overall survival; SBS, Single base substitution.
